# TEOA Promotes Autophagic Cell Death via ROS-Mediated Inhibition of mTOR/p70S6k Signaling Pathway in Pancreatic Cancer Cells

**DOI:** 10.3389/fcell.2021.734818

**Published:** 2021-10-06

**Authors:** Chen Yang, Yanchun Li, Wanye Hu, Xu Wang, Jiayu Hu, Chen Yuan, Chaoting Zhou, Hairui Wang, Jing Du, Ying Wang, Xiangmin Tong

**Affiliations:** ^1^Department of Ultrasound, Zhejiang Provincial People’s Hospital, Affiliated People’s Hospital, Hangzhou Medical College, Hangzhou, China; ^2^Department of Central Laboratory, Affiliated Hangzhou First People’s Hospital, Zhejiang University School of Medicine, Hangzhou, China; ^3^Graduate School, Bengbu Medical College, Bengbu, China; ^4^Laboratory Medicine Center, Zhejiang Provincial People’s Hospital, Affiliated People’s Hospital, Hangzhou Medical College, Hangzhou, China; ^5^School of Laboratory Medicine and Life Science, Wenzhou Medical University, Wenzhou, China; ^6^School of Pharmacy, Zhejiang University of Technology, Hangzhou, China; ^7^Clinical Pharmacy Center, Zhejiang Provincial People’s Hospital, Affiliated People’s Hospital, Hangzhou Medical College, Hangzhou, China

**Keywords:** TEOA, autophagy, pancreatic cancer cells, mTOR, mitochondria injury, ROS

## Abstract

Pancreatic cancer is a common malignant tumor with high mortality, and novel therapeutic options have focused on ameliorating its poor prognosis. TEOA, a traditional Chinese herbal medicine, exhibits anti-inflammatory and anti-cancer activities. Our recent study has shown that TEOA inhibits proliferation and induces DNA damage in diffuse large B-cell lymphoma cells by activating the ROS-mediated p38 MAPK pathway. However, its effects on pancreatic cancer cells remain unknown. In the present study, we evaluated the effects of TEOA on the proliferation, migration of pancreatic cancer cells and explored the possible underlying mechanism of action. We found that TEOA significantly inhibited the proliferation and migration of pancreatic cancer cells in a time- and dose-dependent manner. Mechanistically, TEOA significantly induced mitochondrial dysfunction in PANC1 and SW1990 cells, as evidenced by the collapse of the mitochondrial membrane potential, exhausted ATP level, and excessive accumulation of intracellular ROS. Notably, our further experiments showed that TEOA induced autophagic cell death in pancreatic ductal adenocarcinoma cells by inactivating the ROS-dependent mTOR/p70S6k signaling pathway. More importantly, both pharmacological or genetic blocking of the autophagic flux signal could partly restore the cytotoxicity of TEOA, whereas activation of autophagy by rapamycin or EBSS induced starvation facilitated the cytotoxicity of TEOA. Concomitantly, N-acetylcysteine, a ROS scavenger, abolished the inhibition of the mTOR signaling pathway, thus preventing autophagy and restoring cell viability. Taken together, our results reveal that TEOA can lead to ROS-dependent autophagic cell death of pancreatic cancer cells by inducing mitochondrial dysfunction, which might be a promising therapeutic agent for pancreatic cancer.

## Introduction

Pancreatic cancer is an aggressive malignancy with poor prognosis and limited treatment options and is the seventh leading cause of cancer mortality worldwide (Allemani et al., 2018). Although enormous effort has been made in novel therapeutics, for example, the combination of surgery and adjuvant chemotherapy was shown to effectively improve the prognosis of patients, the 5-year survival rate of patients is still less than 10% (Neoptolemos et al., 2018). Most patients with pancreatic cancer are diagnosed at a late stage, with some even exhibiting distant metastasis, and most patients die within several months. Owing to the high malignancy and poor prognosis of pancreatic cancer, it is imperative to develop more effective and less toxic therapeutic strategies.

Traditional medicinal herbs have long been used in China because of their low toxicity and high activity. Natural compounds derived from medicinal herbs have been demonstrated to be the mainstay of anticancer drug screening. Many FDA-approved anti-cancer drugs have been developed from natural compounds, such as paclitaxel, camptothecin, and podophyllotoxin (Cragg et al., 2009; Newman and Cragg, 2012). Our previous studies also demonstrated that dihydroartemisinin, derived from the Chinese plant *Artemisia annua*, preferentially targeted acute myeloid leukemia cells by inducing ferroptotic cell death while exhibiting low toxicity on normal hematopoietic progenitor cells (Du et al., 2019). Gambogic acid, derived from the Chinese herb *Garcinia hanburyi*, induces T-cell acute lymphoblastic leukemia cell apoptosis by downregulating the β-catenin signaling pathway (Wang et al., 2020).

*Actinidia eriantha* Benth is a traditional Chinese herb belonging to the family *Actinidiaceae*. The antitumor and immunomodulatory activities of *A. eriantha* Benth have been demonstrated in previous studies (Xu et al., 2009; Wu et al., 2017). TEOA (2a,3a,24-*thrihydroxyurs*-12-en-28-*oicacid*) is a pentacyclic triterpenoid isolated from the roots of *A. eriantha* Benth, which exhibits tremendous anti-colon cancer effects both *in vivo* and *in vitro* (Zhang et al., 2017). Another recent study also revealed that TEOA inhibits proliferation and induces DNA damage in diffuse large B-cell lymphoma cells (DLBCL) by activating the ROS-mediated p38 MAPK pathway (Yu et al., 2020). Owing to its high safety and anti-cancer efficiency, we speculated whether TEOA could target pancreatic cancer cells and be developed as a prospective therapeutic agent.

Autophagy is an important process in intracellular substance turnover and metabolism in eukaryotic cells mediated by lysosomes, which is closely related to tumorigenesis and development (Levy et al., 2017). Traditionally, cell autophagy is considered a protective mechanism that promotes cell survival via the stress response (Saha et al., 2018). However, extensive studies have shown that autophagy leads to cell death, called autophagic cell death (ACD) (Shchors et al., 2015). In the current study, we investigated the antitumor activity of TEOA in the human pancreatic cancer cell lines SW1990 and PANC1 and explored the underlying mechanisms. Our results indicated that TEOA effectively inhibited the proliferation and migration of pancreatic cancer cells. In addition, we revealed that TEOA induced the collapse of mitochondrial membrane potential (MMP) and exhausted ATP levels accompanied by the excessive accumulation of intracellular ROS. Notably, our further experiments showed that TEOA induced ACD in pancreatic ductal adenocarcinoma (PDAC) cells by inactivating the mTOR/p70S6k/S6 signaling pathway. Additionally, N-acetylcysteine (NAC) prevented autophagy and restored cell viability by ameliorating ROS-mediated mTOR inactivation. Taken together, our results indicate that TEOA may act as a promising therapeutic agent for pancreatic cancer by inducing ACD.

## Materials and Methods

### Cell Lines and Culture Conditions

Human pancreatic cancer cell lines SW1990 and PANC-1 were purchased from the Cell Bank of the Chinese Academy of Sciences (Shanghai, China). The human normal pancreatic cell line HPDE6-C7 was obtained from BLUEFBIO biological company (Shanghai, China). All cell lines were cultured in Dulbecco’s modified Eagle’s medium (DMEM, Hyclone, United States), supplemented with 10% fetal bovine serum (FBS, Gibco, United States), 100 U/mL penicillin, and 100 μg/mL streptomycin (Solarbio, China), and incubated at 37°C in a humidified atmosphere with 5% CO_2_.

### Reagents and Antibodies

TEOA was obtained from college of pharmacy, Zhejiang University (Zhejiang, China) and dissolved in DMSO. N-acetylcysteine (NAC), Chloroquine (CQ), 3-Methyladenine (3MA) and Rapamycin were purchased from Medchem Express (MCE, United States). DAPI was purchased from Beyotime (Shanghai, China). The following primary antibodies were used: anti-GAPDH (Abcam ab181602) (1:2,000), anti-Catalase (Abcam ab76024) (1:1,000), anti-MnSOD (Abcam ab68155) (1:2,000), anti-LC3 (Sigma L7543) (1:1,000), anti-p-mTOR (Abcam ab109268) (1:2,000), anti-mTOR (Abcam ab134903) (1:1,000), anti-p-p70S6K (Abcam ab131436) (1:1,000), anti-p70S6K (Abcam ab32529) (1:1,000), anti-p-S6 (CST 4858S) (1:2,000), anti-S6 (CST 2217s) (1:1,000), anti-p-4EBP1 (CST 9459S) (1:1,000), anti-4EBP 1 (CST 9644S) (1:1,000), anti-ATG5 (Abcam ab68155) (1:2,000). The related HRP-conjugated secondary antibody was purchased from Beyotime (Shanghai, China).

### Cell Viability Assays

SW1990, PANC-1 and HPDE6-C7 cells (2 × 10^4^ per well) were plated in 96-well plates (NEST Biotechnology). Cells were treated with TEOA (0–60 μM) for 12 h, 24 h at 37°C. The viability of cells was detected by the Cell Counting Kit 8 (CCK-8) assay. The optical density of each well was measured at 450 nm with a microplate reader (Thermo, United States).

### Lactate Dehydrogenase (LDH) Assay

SW1990, PANC1 and HPDE6-C7 cells (2 × 10^4^ per well) were plated in 96-well plates. After being treated with TEOA (0–60 μM) for 12 h at 37°C, LDH activities of TEOA treated cells were measured by LDH cytotoxicity assay kit (Beyotime, Shanghai, China) according to the manufacturer’s instructions.

### Calcein/PI Cell Viability/Cytotoxicity Assay

To further detect the effect of TEOA on normal cells and pancreatic cancer cells, SW1990 and HPDE6-C7 cells (2 × 10^4^ per well) were plated in 96-well plates. After 12 h, cells were treated with TEOA (0–50 μM) for the indicated time at 37°C. Then the living cells and the dead cells were visualized by Calcein/PI cell viability/cytotoxicity assay kit (Beyotime, Shanghai, China) and photographed under EVOS M7000 (Thermo Fisher Scientific, United States).

### Observation of Cell Morphology

SW1990 and PANC1 cells were seeded at 6 × 10^5^ cells per well in 6-well plates overnight and then treated with vehicle (DMSO) or TEOA at various concentrations and times. Then the cellular morphology change was observed under the microscope and photographed (Nikon, Japan).

### 5-Ethynyl-2’-Deoxyuridine (EdU) Assay

5-ethynyl-2’-deoxyuridine (EdU) staining was performed by a commercial kit (Beyotime, China) according to the manufacturer’s instructions. Briefly, treated cells were incubated with 10 μM EdU for 2 h at 37°C and then fixed with 4% paraformaldehyde for 15 min at 37°C. Subsequently, the cells were permeabilized with 0.3% Triton X for 15 min at room temperature. After washing twice with PBS, the cells were incubated with a click-reaction cocktail in the dark for 30 min. DNA was stained with DAPI for 5 min and visualized under confocal microscopy (Leica, Germany).

### Anchorage-Dependent Colony Formation

For the colony assay, 1 × 10^3^ cells were seeded in 12-well plates and cultured with TEOA for 7 days. Cells were fixed with methanol for 15 min before staining with 0.5% crystal violet (Beyotime, Shanghai, China). Colonies were counted using ImageJ and then normalized to the number of colonies in the control group.

### Transwell Assay

For the cell migration assay, cells (4 × 10^4^ cells/well) were incubated in the upper transwell chambers (Corning, United States) with the indicated concentration of TEOA dissolved in serum-free DMEM, and DMEM containing 10% FBS was added to the lower chamber as an attractant. After 24 h, the cells on the lower membrane were fixed with 4% paraformaldehyde for 15 min and stained with 0.1% crystal violet. Finally, the cells were photographed under a microscope (Nikon, Japan), and the number of cells was quantified.

### Cell Scratch Assay

To evaluate the effect of TEOA on cell migration *in vitro*, we performed a scratch assay. Cells were seeded onto a 6-well plate at a density of 8 × 10^5^ cells/well. A sterile 10 μL pipette tip was used to make a straight scratch line on the confluent cell monolayer when the cells had nearly reached confluency. The medium was replaced with 2% FBS medium containing TEOA. Cell scratches were visualized at 0, 24, and 48 h and imaged by inverted microscope (Nikon, Japan).

### Confocal Microscopy

Cells were seeded in a confocal dish (NEST, China) and treated with TEOA at the designated concentrations, then stained with Mitotracker, Mitosox (Thermo Fisher Scientific, United States), Magic Red cathepsin B (Immunochemistry Technologies) or DCFH-DA (Sigma-Aldrich, St. Louis, MO, United States) for 30 min in the dark. Nuclei were co-stained with DAPI (10 μg/mL). The cells were viewed and imaged under a confocal microscope (Leica, Germany).

### Measurement of Mitochondria Membrane Potential (MMP)

Fluorescent probe Tetramethyl Rhodamine Methyl Ester (TMRM, Thermo Fisher Scientific, Waltham, MA, United States) was used to evaluate the MMP. Cells were seeded in 6-well plate (6 × 10^5^ cells per well) overnight and treated with TEOA for 8 h. Then the cells were stained with TMRM (200 nM) for 30 min in the dark, and followed by flow cytometry detection.

### Measurement of Intracellular ATP Levels

To measure the intracellular ATP levels, cells were seeded onto 6-well plates (6 × 10^5^ cells per well) and then treated with TEOA at different concentrations for 8 h. Intracellular ATP levels were measured using ATP assay kits in accordance with the manufacturer’s instructions (Beyotime, China).

### Determination of Intracellular ROS Production

The fluorescent probe DCFH-DA (Sigma-Aldrich, St. Louis, MO, United States) was used to measure intracellular ROS production. PANC-1 and SW1990 cells were seeded onto 6-well plates (6 × 10^5^ cells per well) and cultured for 12 h. Subsequently, the cells were directly exposed to TEOA for 8 h at the indicated concentrations and cotreated with or without NAC (0.5 mM). Treated cells were harvested, and intracellular ROS levels were determined by flow cytometry or microplate spectrophotometer (BioTek, United States) according to the manufacturer’s instructions.

### Western Blotting

Cells were seeded onto a 6-well plate (7 × 10^5^ cells per well) and cultured for 12 h. The cells were treated as designed for 8 h. After being collected and lysed with RIPA buffer containing complete protease and phosphatase inhibitor (Thermo, Waltham, MA, United States) for 10 min, the lysates were centrifuged at 12,000 × *g* for 15 min at 4°C. BCA Protein Assay Kit (Beyotime, China) was used to quantify the protein concentration in the supernatant. Equal amounts of proteins were separated using polyacrylamide gel electrophoresis and subsequently transferred to a polyvinylidene fluoride membrane (Bio-Rad, United States). The membrane was blocked with 5% skim milk in Tris-buffered saline with 0.1% Tween-20 (TBST) for 2 h and incubated with primary antibodies overnight at 4°C. The next day, the membrane was washed three times with TBST and incubated with the appropriate secondary antibodies for 1 h at room temperature. Finally, the immunoblots were visualized using an enhanced chemiluminescence system (Bio-Rad, United States). ImageJ was used to quantify the band intensities.

### mRFP-GFP-LC3 Imaging

We used mRFP-GFP-LC3 adenovirus (Hanbio, Shanghai, China) to further confirm the autophagic flux. Briefly, mRFP-GFP-LC3 adenovirus was transfected into SW1990 cells, in accordance with the manufacturer’s instructions. After 36 h, transfected SW1990 cells were treated as described, followed by staining with DAPI (10 μg/mL) at 37°C for 15 min. Autophagic vesicles were observed and photographed under a confocal fluorescence microscope.

### Transmission Electron Microscope (TEM)

Transmission electron microscope was used to determine the occurrence of autophagy. SW1990 cells were first fixed with 2.5% glutaraldehyde for 4 h and postfixed with 1% OsO_4_ in phosphate buffer for 2 h. Then the samples were dehydrated by a graded series of ethanol (30, 50, 70 and 80%) alcohol. Subsequently, the specimens were sectioned in LEICA EM UC7 ultratome and stained by uranyl acetate and alkaline lead citrate for 5 min, respectively. The ultrastructural characteristics of autophagy in different treatment groups were observed by TEM (Hitachi, H-7650, Japan).

### Construction of *ATG5* Knockdown Vector

For knockdown of *ATG5*, target shRNA sequences were subcloned into pLVX-shRNA lentivirus vector (Takara, Japan). The shRNA sequences for *ATG5* knockdown were shown below. sh*ATG5* forward sequence: GATCCGCCTGAACAGAATCATCCTTAATTCA AGAGATTAAGGATGATTCTGTTCAGGTTTTTTG, shATG5 reverse sequence: AATTCAAAAAACCTGAACAGAATCA TCCTTAATCTCTTGAATTAAGGATGATTCTGTTCAGGCG. The recombinant lentiviral plasmids were verified by sequencing. The empty vector was used as a control.

### Apoptosis Evaluation

Cell apoptosis was detected by Annexin V-FITC/PI apoptosis kit (MultiSciences, China) with a flow cytometry. Cells were seeded in 6-well plates and cultured with TEOA at indicated concentrations for 12 h. The cells were then harvested and stained with Annexin V-FITC/PI for 5 min, then followed by flow cytometry.

### Statistical Analyses

Data are exhibited as mean ± standard deviation (mean ± SD) from three independent experiments. IC50s were calculated using non-linear regression by GraphPad Prism. Log dose-response curves were plotted using the non-linear regression (Dose-response Inhibition-log (agonist) vs. response module). The differences between two groups were detected using the Student’s *t* test. The differences between multiple groups were detected using the analysis of variance (ANOVA). *p* < 0.05 was defined as statistically significant difference. ^★^*p* < 0.05, ^★★^*p* < 0.01, ^★★★^*p* < 0.001.

## Results

### TEOA Exerted Anti-cancer Cytotoxicity on Human Pancreatic Cancer Cells

**FIGURE 1:**
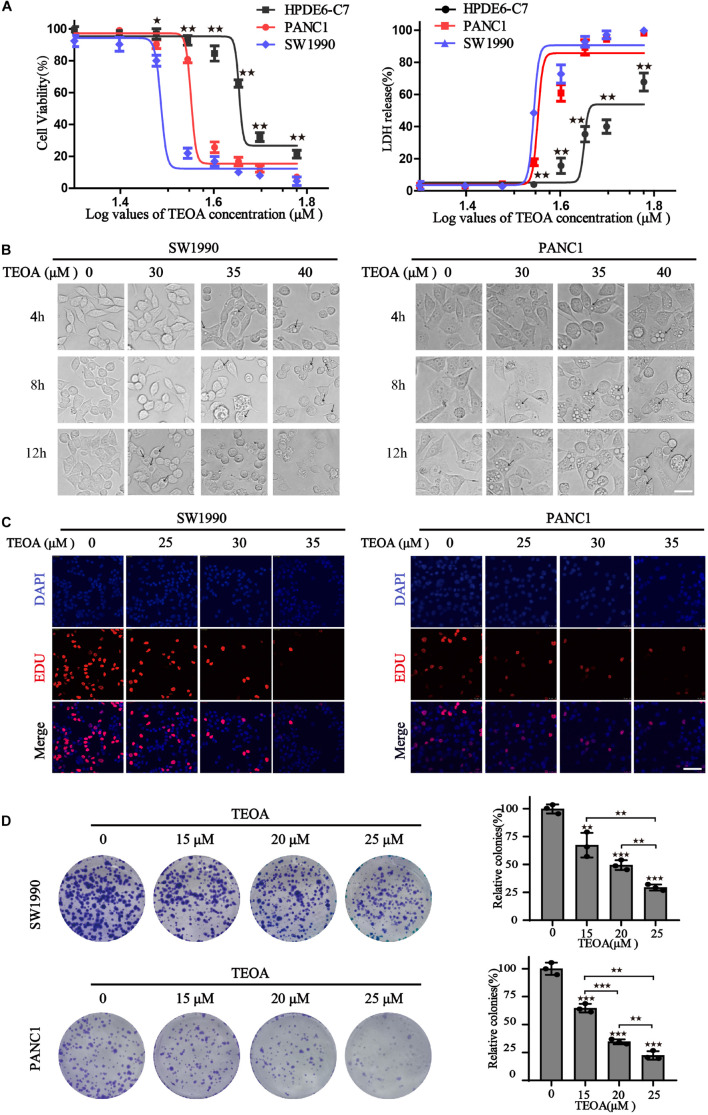
TEOA induced relative selective cytotoxicity of human pancreatic cancer cells. **(A)** HPDE6-C7, SW1990, and PANC1 cells were treated with various concentrations of TEOA (0–60 μM) for 12 h. The cell viability was detected using CCK-8 assays (left). LDH activities of drug-treated cells were measured by LDH cytotoxicity assay (right). **(B)** Cells were treated with 30, 35, 40 μM TEOA for 4 h, 8 h, 12 h and photographed by microscope. Scale bars 20 μm. The arrows were used to mark the vacuolation changes. **(C)** Cells were treated with TEOA at 0, 25, 30, 35 μM and cell proliferation was detected by EdU assay after 12 h. **(D)** Cell proliferation ability with long-term TEOA treatment was estimated by the colony formation assay, and colonies were analyzed on the right (^★^*p* < 0.05; ^★★^*p* < 0.01; ^★★★^*p* < 0.001, between groups).

To evaluate the selective anti-cancer activity of TEOA in pancreatic cancer cells, the cytotoxic effects of TEOA on two pancreatic cancer cell lines (PANC1, SW1990) and normal pancreatic cell line HPDE6-C7 were detected using the CCK-8 assay and LDH release assay. CCK-8 assay showed that TEOA significantly reduced the viability of cancer cells in a dose-dependent manner (*p* < 0.01). The IC-50s of TEOA at 12 h in the normal pancreatic cell line and pancreatic cancer cell lines were 47.84 μM (HPDEC-C7), 38.02 μM (PANC1), and 32.71 μM (SW1990), respectively (Figure 1A). At 24 h, TEOA also showed preferential tumor cytotoxicity (Supplementary Figure 1A). Similarly, LDH release from TEOA-treated PANC1 and SW1990 cells were obviously higher than TEOA-treated HPDE6-C7 cells (Figure 1A). In Supplementary Figure 1B, Calcein/PI cell viability/cytotoxicity assay further confirmed that TEOA preferentially targeted pancreatic cancer cells, while sparing low toxicity on non-malignant cells.

In addition, changes in cell morphology were monitored using a light microscope. With the increase in time and drug concentration, severe vacuolation was observed in the cytoplasm and the cancer cells tended to be fragmented (Figure 1B). EdU staining and colony formation assay were also performed to evaluate the inhibitory effects of TEOA on cell proliferation. As shown in Figure 1C, with an increase in TEOA concentration, the number of EdU-positive cells decreased gradually. Consistently, Figure 1D shows a concentration-dependent decrease in colony formation. Taken together, our findings indicated that TEOA exhibited an efficient cytotoxic effect on pancreatic cancer cells.

### TEOA Suppressed Migration of SW1990 and PANC1 Cells

**FIGURE 2:**
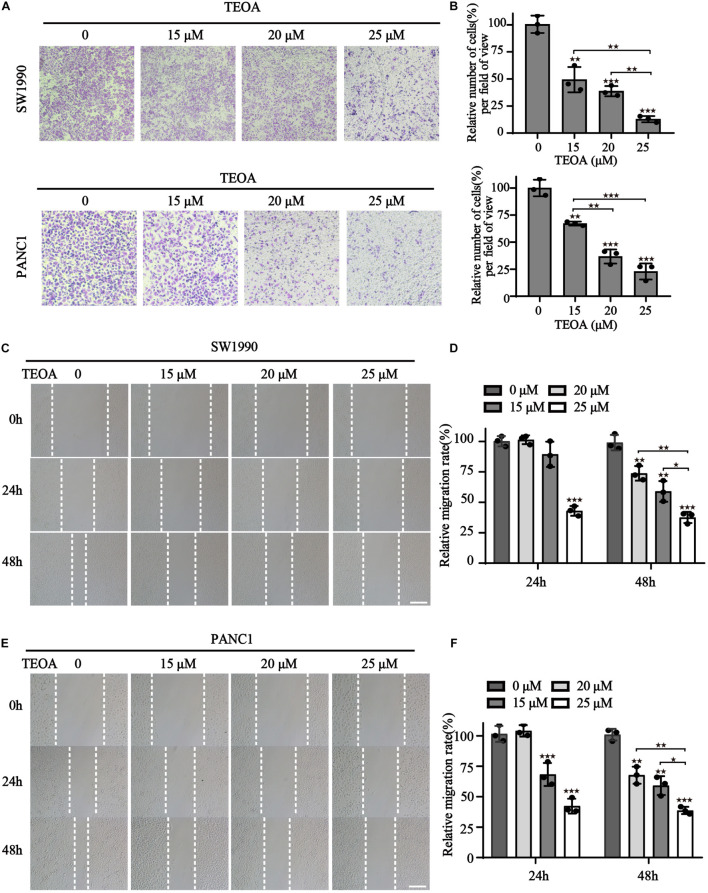
TEOA suppressed migration of SW1990 and PANC1 cells. **(A)** Migration of PANC1 and SW1990 cells treated with 0, 15, 20, 25 μM TEOA for 24 h was determined using the transwell assay. **(B)** Statistical analysis of the migration cells. **(C–F)** Scratch wound healing assay was used to detect the migration capability of cells with the indicated concentrations of TEOA and representative microscopic images were showing in the **(C,E)**. Relative migration distance was quantified and shown on the right (^★^*p* < 0.05; ^★★^*p* < 0.01; ^★★★^*p* < 0.001, versus control).

In addition to proliferation, migration of cancer cells is another important hallmark of cancer progression. Therefore, we performed cell transwell assays and scratch tests to determine the influence of TEOA on cell migration. The results showed that TEOA significantly suppressed the migration of pancreatic cancer cells in a dose-dependent manner. The relative migration rate (%) decreased to 49.34 ± 11.69% (*p* < 0.01), 38.69 ± 4.70% (*p* < 0.001), and 12.87 ± 2.89% (*p* < 0.001) after treatment with 15, 20, and 25 μM TEOA, respectively. In PANC1 cells, the relative invasion rate (%) decreased to 65.18 ± 1.84% (*p* < 0.01), 36.72 ± 6.50% (*p* < 0.001), and 22.88 ± 7.44% (*p* < 0.001), respectively (Figures 2A,B). Consistently, the scratching test also exhibits a similar result (Figures 2C,E). The relative migration rate of SW1990 cells incubated with 25 μM TEOA decreased to 37.54 ± 4.57% compared with that of the control group at 48 h (*p* < 0.01) (Figure 2D). The relative migration rate of PANC1 cells treated with 25 μM TEOA decreased to 38.67 ± 2.99% (*p* < 0.01) (Figure 2F). Similar trends were observed at 24 h. On the contrary, TEOA exhibits lesser effects on the migration of HPDE6-C7 cells (Supplementary Figure 2). These results revealed that TEOA suppressed the migration of SW1990 and PANC1 cells.

### TEOA Induced Mitochondrial Dysfunction and Oxidation Stress of Pancreatic Cancer Cells

**FIGURE 3:**
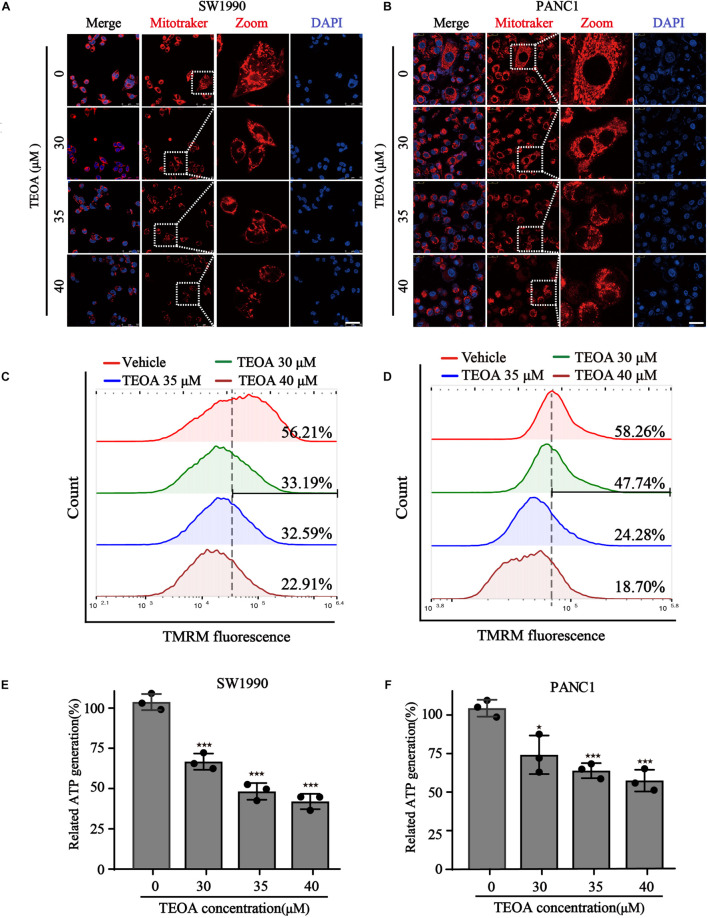
TEOA induced mitochondrial dysfunction of pancreatic cancer cells. **(A,B)** To observe the morphology of mitochondria, SW1990 and PANC1 cells treated with the indicated concentration of TEOA for 8 h and stained with Mitotracker probe (200 nM), DAPI (10 μg/ml), and then photographed by confocal laser microscope. Scale bars 50 μm. **(C,D)** To assess the changes of mitochondria membrane potential, TEOA treated SW1990 cells and PANC1 cells were stained with TMRM probe for 30 min and followed by flow cytometry measurement. **(E,F)** SW1990 and PANC1 cells were treated with the indicated concentration of TEOA, then ATP levels were measured. ATP levels of TEOA treated PANC1 cells were measured (^★^*p* < 0.05; ^★★^*p* < 0.01; ^★★★^*p* < 0.001, versus control).

Mitochondria coordinate a large fraction of metabolic, energetic, and physiological processes, and their functional integrity is crucial for the proliferation, migration, and metabolism of cancer cells (Picard et al., 2018). We verified whether TEOA could impair mitochondrial homeostasis in pancreatic cancer cells. We first focused on mitochondrial morphology, an early event of mitochondrial dysfunction, which was visualized by the MitoTracker Red probe and observed under a confocal fluorescence microscope. Compared with the healthy tubular network structure of mitochondria in the control group, TEOA induced concentration-dependent changes in mitochondrial morphology, characterized by reduced cristae and fragmentation, in SW1990 and PANC1 cells (Figures 3A,B). In addition to morphological changes, MMP, another reliable indicator to evaluate mitochondrial functional damage, was monitored by the fluorescent dye TMRM (Kang et al., 2017). The results showed that the MMP of two pancreatic cancer cells was significantly decreased after treated with different concentrations of TEOA (Figures 3C,D). Because MMP is maintained through mitochondrial oxidative phosphorylation, we detected the ATP turnover. As expected, ATP levels in pancreatic cancer cells treated with TEOA showed a similar downward trend (Figures 3E,F). In summary, our results emphasize that TEOA treatment impairs mitochondrial function in pancreatic cancer cells.

**FIGURE 4:**
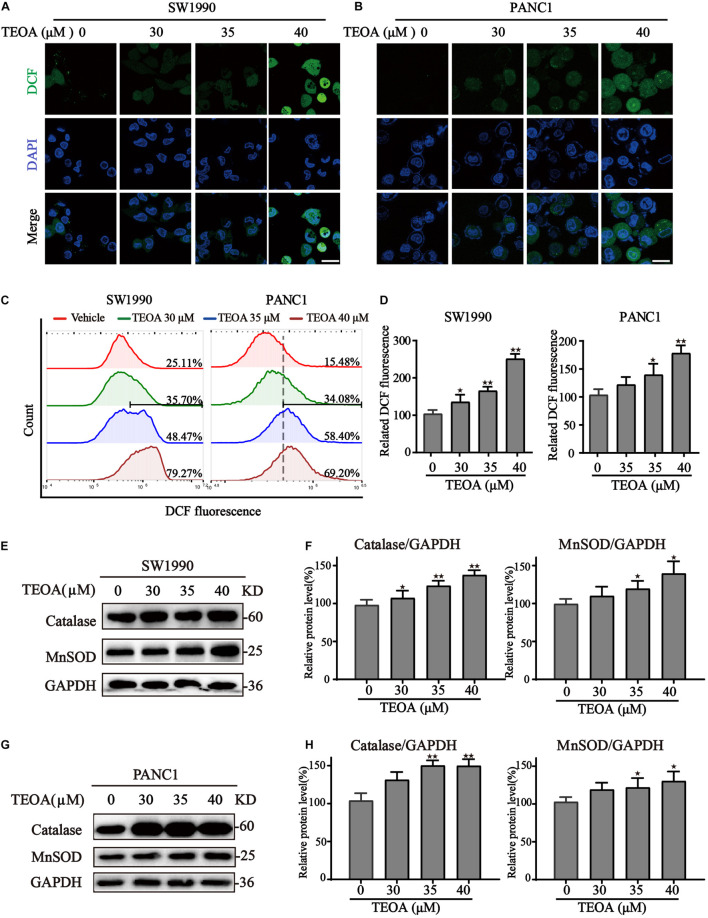
TEOA induced ROS accumulation and oxidative stress in pancreatic cancer cells. **(A–D)** To assess intracellular ROS production, TEOA treated SW1990 and PANC1 cells were loaded with DCFH-DA probe for 30 min followed by confocal laser microscope (**A,B**, Scale bars: 25 μm) and flow cytometry measurement **(C,D)**. **(E)** SW1990 cells were treated with TEOA at indicated concentrations for 8 h, and expression of oxidative stress-related proteins catalase and MnSOD were detected by western blot. GAPDH was used as a loading control. **(F)** Catalase and MnSOD were quantified by normalizing to GAPDH. **(G,H)** Similarly, catalase and MnSOD expression of TEOA-treated PANC1 cells were determined by western blot, and the quantitative results were shown on the right (^★^*p* < 0.05; ^★★^*p* < 0.01, versus control).

Once mitochondrial dysfunction occurs, large quantities of ROS leak from the electron transport chain, resulting in oxidative stress (Panel et al., 2018). The DCFH-DA probe was then used to detect ROS levels in cells treated with TEOA. Confocal images revealed a significant increase of ROS levels in PANC1 and SW1990 cells treated with ascending dose of TEOA (Figures 4A,B). Notably, we could observe a significant accumulation of mitochondrial ROS after the treatment of TEOA, whereas TEOA did not induce significant oxidative stress in HPDE6-C7 cells (Supplementary Figure 3). Consistent with the results of fluorescent confocal microscopy, flow cytometry analysis of DCF also demonstrated that TEOA facilitated the accumulation of cellular ROS (*p* < 0.05; Figures 4C,D). Furthermore, the expression of oxidative stress-related proteins after TEOA treatment was determined by western blot. The results indicated that antioxidant molecules, including MnSOD and catalase, were upregulated and activated as an adaptive response to maintain tumor cell survival (Figures 4E–H). Furthermore, we found that co-treatment with the ROS scavenger NAC could not restore the MMP, which confirmed the upstream position of mitochondrial dysfunction (Supplementary Figure 4). These results revealed that TEOA impaired mitochondrial homeostasis and thus led to an oxidative stress response.

### TEOA Induced Autophagy via Inhibiting the AMPK/mTOR/p70s6k/S6 Signaling Pathway

**FIGURE 5:**
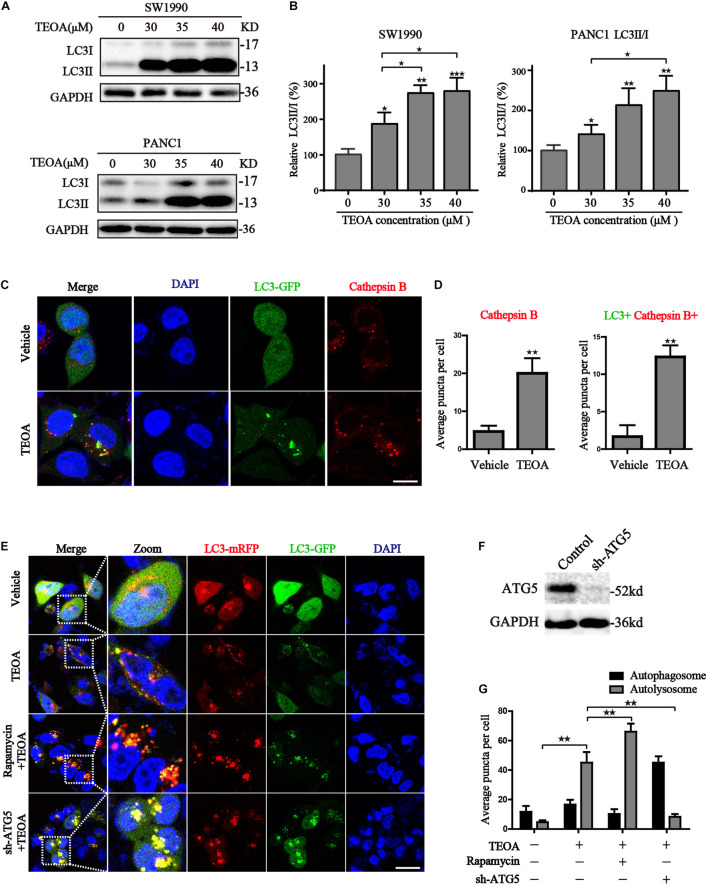
TEOA induced autophagy in pancreatic cancer cells. **(A)** SW1990 and PANC1 cells were treated with TEOA at the indicated concentration of TEOA for 8 h and the ratio of LC3I and LC3II were detected by western blot. **(B)** The quantitations of LC3II/I were shown on the right. **(C)** SW1990 cells transfected with GFP-LC3 were treated with TEOA for 8 h and then stained with cathepsins, the changes of fluorescence were photographed by confocal microscopy. **(D)** Quantitative analysis for the Cathepsins + /LC3+ Cathepsins+ puncta of cells. **(E)** Wide type and ATG5 shRNA-transfected SW1990 cells were treated with TEOA alone or combined with Rapamycin (0.4 μM) for 8 h following transfection with GFP-mRFP-LC3. Representative pictures were recorded using confocal microscopy, scale bar: 25 μm. **(F)** SW1990 cells were transfected with control shRNA and ATG5 shRNA, respectively, and western blot was used to confirm the knock-down effects of ATG5. **(G)** Quantitative analysis for the autophagosomes and autolysosome (^★^*p* < 0.05; ^★★^*p* < 0.01; ^★★★^*p* < 0.001, versus control).

Mitochondria plays an indispensable role in maintaining the normal physiological processes in cells (Pustylnikov et al., 2018). ROS accumulation due to mitochondrial dysfunction results in the initiation of cell autophagy (Hatchi et al., 2011; Yang et al., 2019). Hence, we measured the levels of autophagy-related proteins in TEOA-treated pancreatic cells to investigate whether TEOA induces autophagy. LC3 is a classical autophagy-related protein, and the conversion of LC3-I to LC3-II is a reliable marker of autophagy initiation. We found that TEOA significantly induced the conversion of LC3, while did not exhibit significant effects on the ratio of LC3II/I in HPDE6-C7 cells (Figures 5A,B; Supplementary Figure 5). The activation of autophagy is accompanied by the formation of autophagosome and the activation of lysosome. Therefore, we used Ad-GFP-LC3B combined with lysosome staining to verify the occurrence of autophagy. As shown in Figure 5C, GFP-LC3B fluorescence is diffusely distributed in the cytoplasm in the basal conditions. After the treatment of TEOA, GFP-LC3B aggregated on autophagosome membrane and co-stained with cathepsin B to form the autolysosome (Figure 5D). Our TEM results also revealed the occurrence of autophagy in TEOA-treated SW1990 cells (Supplementary Figure 6).

In addition, we further verified the autophagic flux using a tandem-tagged mRFP-GFP-LC3 adenovirus and evaluated the results by confocal microscopy. The appearance of red vesicles after TEOA treatment marked the autolysosomal event that occurred after the fusion of lysosomes with autophagosomes. When the autophagic flux is disrupted during the fusion of lysosomes with autophagosomes, autophagosomes are present as yellow vesicles. Compared with TEOA-treated alone, combination of rapamycin further enhanced autophagic flux (*p* < 0.01). While, *ATG5* knockdown effectively inhibited TEOA-induced autophagy (*p* < 0.01) (Figures 5E,G). The knockdown efficiency of *ATG5* in SW1990 cells was evidenced by the western blot assay (Figure 5F). Therefore, we can conclude that TEOA is capable of promoting autophagic flux.

**FIGURE 6:**
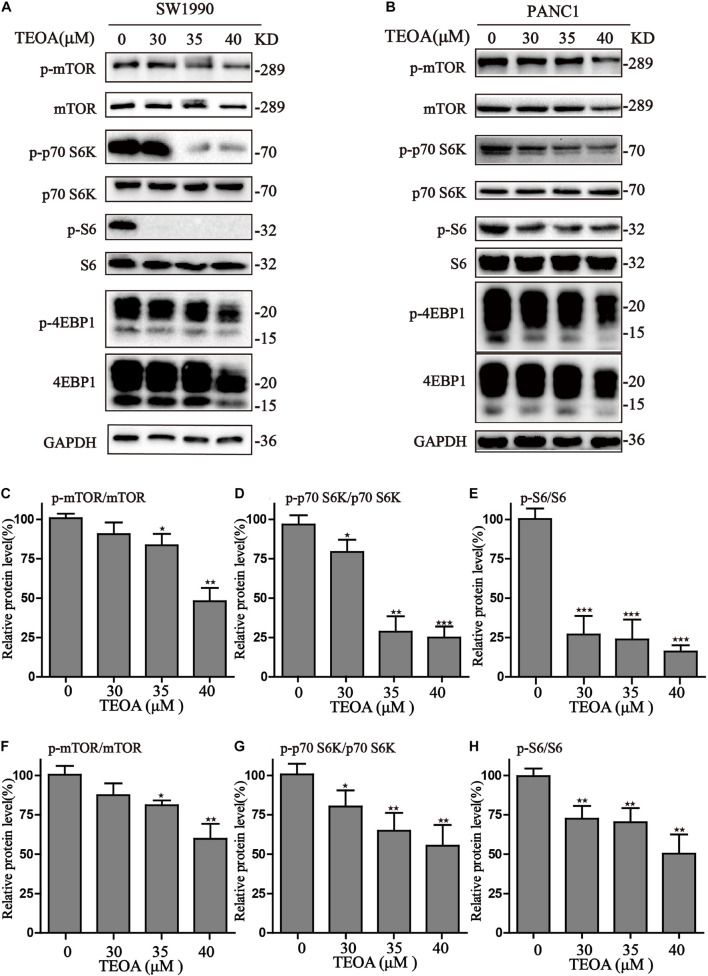
TEOA induced autophagy via inhibiting mTOR/p70s6k/S6 signaling pathway in pancreatic cancer cells. **(A,B)** After exposure to TEOA for 8 h, SW1990 and PANC1 cells were collected to measure the signaling pathway of mTOR/p70s6k/S6, GAPDH used as a loading control. **(C–E)** Relative quantitative analysis of SW1990 was shown. **(F–H)** Relative quantitative analysis of PANC1 was shown (^★^*p* < 0.05; ^★★^*p* < 0.01; ^★★★^*p* < 0.001, versus control).

To further demonstrate the mechanisms of autophagy induced by TEOA, we analyzed the AMPK/mTOR/p70s6k/S6 signaling pathway, which was previously demonstrated to play a role in the initiation of autophagy. As shown in Figure 6, TEOA decreased the phosphorylation of mTOR and its downstream proteins, including p70s6k, S6, and 4EBP1. Furthermore, we observed the increase of p-AMPK in TEOA treated pancreatic cancer cells (Supplementary Figure 7). Collectively, TEOA treatment significantly affecting the AMPK/mTOR/p70s6k/S6 signaling pathway, thus increasing the autophagic flux, which might play a critical role in the cytotoxicity of TEOA.

### Role of Autophagy in TEOA-Induced Cytotoxicity in Pancreatic Ductal Adenocarcinoma Cells

**FIGURE 7:**
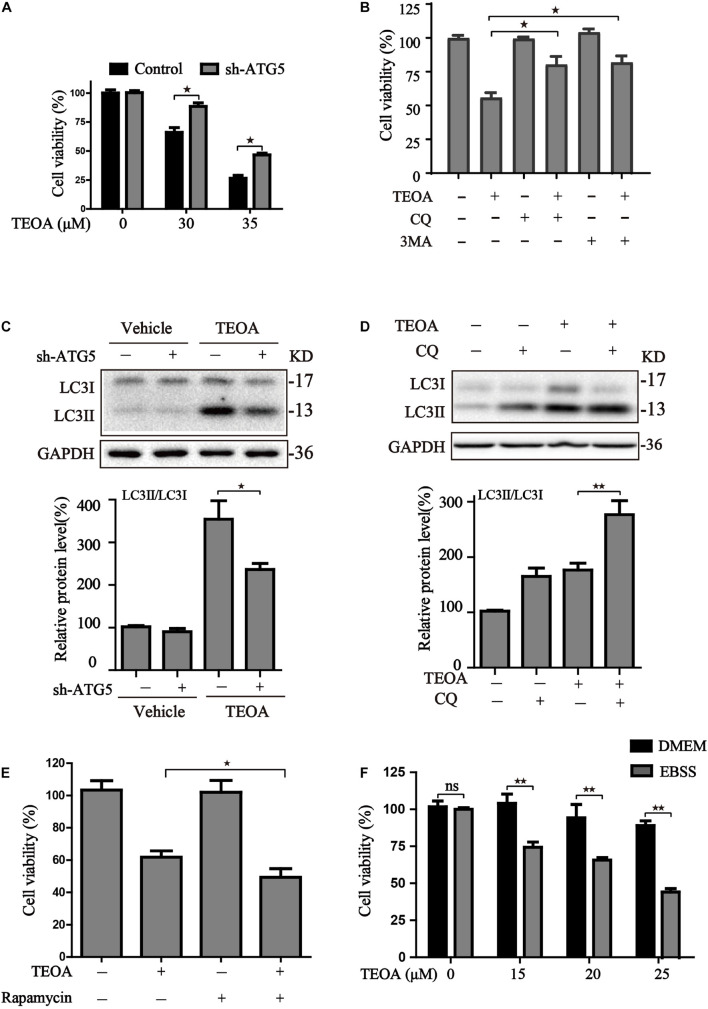
The effects of autophagy activity on TEOA-induced cytotoxicity. **(A)** Control and *ATG5* shRNA-transfected SW1990 cells were treated with 0, 30, 35 μM TEOA. The cell viability was evaluated using CCK-8 assay after 12 h. **(B)** SW1990 cells were treated with TEOA (32 μM) alone or combined with CQ (25 μM), 3MA (5 mM), respectively. CCK-8 test was used to assess cell viability. **(C)** Control and *ATG5* shRNA-transfected SW1990 cells were treated with TEOA (45 μM) for 8 h, **(D)** SW1990 cells were treated with TEOA alone or combined with CQ (25 μM), respectively, and expression of LC3 were detected by western blot, GAPDH was used as a loading control. **(E)** Cells were exposed to TEOA (30 μM) with or without rapamycin (0.4 μM) for 12 h, and then cell viability was measured using the CCK-8 assay. **(F)** SW1990 cells were pretreated with or without EBSS for 4 h and then incubated with TEOA for 12 h. Cell viability was measured by CCK-8 assay (^★^*p* < 0.05; ^★★^*p* < 0.01, versus control).

Autophagy is a catabolic process that plays a central role in maintaining cell metabolism and renewal. In cancer cells, autophagy has a dual role, acting as an adaptive stress response to maintain tumor cell survival, but excessive stimulation of autophagy can be lethal for cancer cells owing to the excess elimination of cytoplasmic content. To confirm the role of TEOA-induced autophagic flux in PDAC cells, we silenced *ATG5* using lentivirus-mediated shRNA to interfere with autophagy and evaluated the cytotoxicity of TEOA. The results of western blotting and confocal microscopy confirmed that *ATG5* knockdown successfully inhibited TEOA-induced autophagy (Figures 5G, 7C). CCK-8 assay showed that the cytotoxic effect of TEOA was partially abrogated by *ATG5* knockdown (Figure 7A). In addition, the autophagosome formation inhibitor (early autophagy inhibitor, 3MA) and autophagosome-lysosome fusion inhibitor (late autophagy inhibitor, CQ) were utilized to confirm these findings. CCK-8 assay showed that both of them partly abrogated the decrease in cell viability following treatment with TEOA (Figure 7B). The autophagy inhibitory efficiency of CQ was also confirmed by western blotting (Figure 7D). We next aimed to determine whether enhanced activation of autophagy exerted a synthetic effect with TEOA and provided different lines of evidence to support the cytotoxicity model. Both administration of rapamycin and EBSS induced starvation sensitized the anticancer activity of TEOA in SW1990 cells (Figures 7E,F). Collectively, these data from both pharmacological and genetic approaches suggest that excessive activation of autophagy acts synergistically with TEOA and renders PDAC cells vulnerable to death, whereas inhibition of autophagy can partly abolish the cytotoxicity of TEOA.

### N-Acetylcysteine Dampened the Cytotoxicity of TEOA via Restoring mTOR/p70s6k Autophagy Signaling Pathway

**FIGURE 8:**
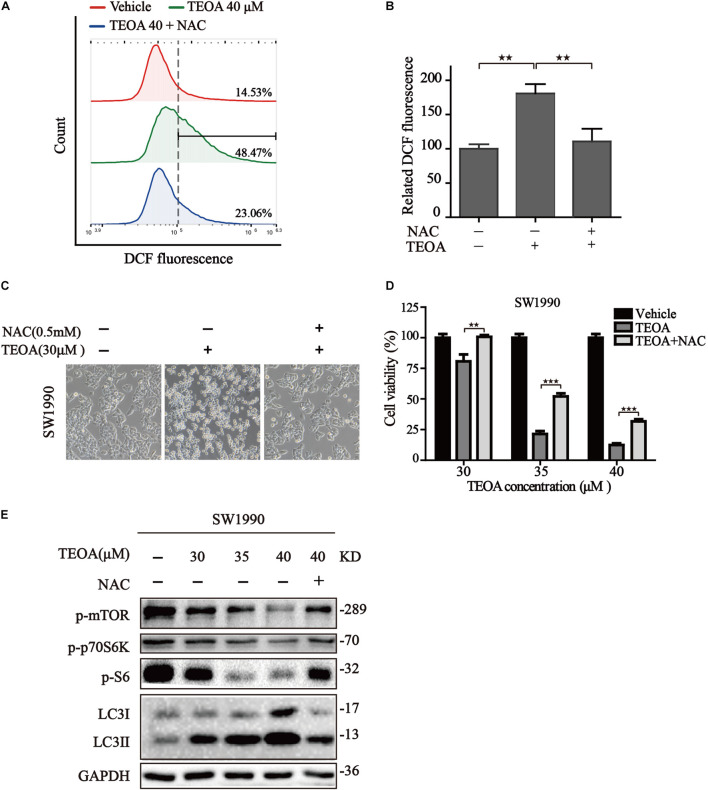
NAC reduced the cytotoxicity of TEOA and inhibited autophagy by restoring mTOR/p70S6k signaling pathway in SW1990 cells. **(A)** SW1990 cells were treated with TEOA alone or in combination with 0.5 mM NAC, and then ROS levels were measured by flow cytometry after 8 h. **(B)** Statistical analysis of relative ROS level and the results were presented as the mean ± SD from three independent experiments. **(C)** SW1990 cells were treated with TEOA alone or in combination with 0.5 mM NAC. After 12 h, images were captured using optical microscope (20×). **(D)** SW1990 cells were treated with TEOA at various concentrations alone or in combination with 0.5 mM NAC for 12 h and cell viability was detected by CCK-8 assay. **(E)** After incubating with individual TEOA or combination with NAC for 8 h, protein expression of mTOR / p70s6k signaling pathway was determined by western blot (^★^*p* < 0.05; ^★★^*p* < 0.01; ^★★★^*p* < 0.001, versus control).

The above mentioned results confirmed that ROS accumulation oriented from mitochondrial dysfunction acts upstream of autophagosome formation, leading to the suppression of mTOR and activating of autophagy. Therefore, it is reasonable to assume that TEOA induced ACD could be mitigated by the inhibition of ROS production. The commonly used ROS scavenger NAC was selected for further study. We found that NAC could notably reverse the oxidative stress induced by TEOA (Figures 8A,B). Furthermore, we examined the changes in morphological features and cell viability following treatment with TEOA in the presence or absence of NAC. As expected, both cell morphology features and cell viability assays validated that coincubation with NAC alleviated cell death induced by TEOA (Figures 8C,D). We further performed western immunoblotting and found that NAC cotreatment restored TEOA-induced mTOR/p70s6k pathway inhibition and alleviated the conversion of LC3 (Figure 8E; Supplementary Figures 8, 9). Collectively, our results confirmed that NAC attenuated excessive activation of autophagy by restoring the mTOR signaling pathway and alleviating ACD induced by TEOA treatment.

## Discussion

**FIGURE 9:**
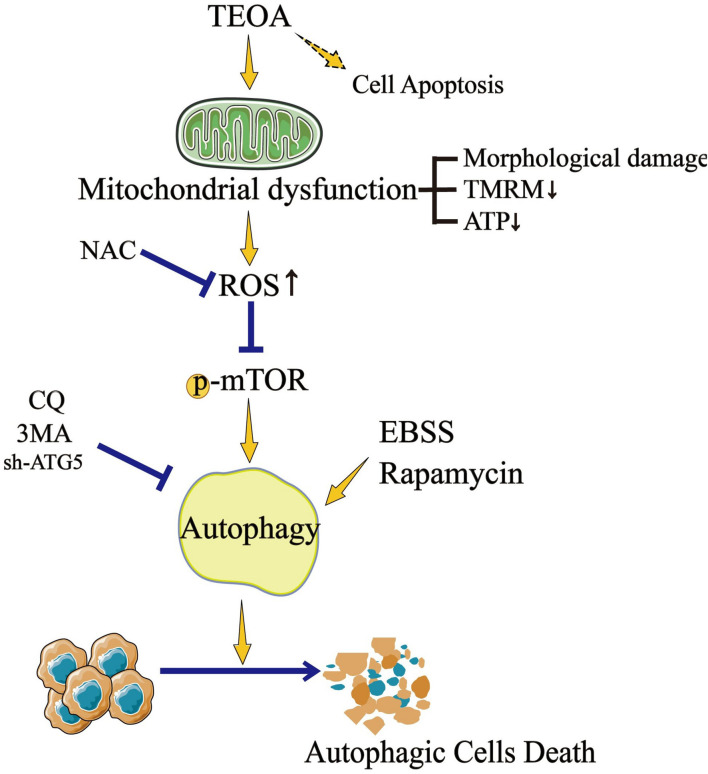
TEOA promotes autophagic cell death via ROS-mediated Inhibition of mTOR signaling pathway.

Traditional herbal medicines are an important source of anti-tumor drugs (Qian et al., 2019). To date, a variety of drug monomers derived from traditional herbal medicines have shown extraordinary anticancer activities through different mechanisms (Zhang et al., 2019). For example, DHA inhibits the proliferation of leukemia cells by inducing ferroptosis (Du et al., 2019). Ovatodiolide suppresses the generation of colon cancer stem cells by reducing cancer stem cell-derived exosomal cargoes (Huang et al., 2020). TEOA is a pentacyclic triterpenoid isolated from the roots of *A. eriantha* Benth, which was reported to induce autophagy in colon cancer cells and DNA damage in malignant lymphoma (Zhang et al., 2017; Yu et al., 2020). Pancreatic cancer is a type of digestive tract malignant tumor with high mortality, however, there is still a lack of effective drugs to improve the prognosis (McGuigan et al., 2018). The application of TEOA may provide new possibilities for the treatment of pancreatic cancer. In the current study, for the first time, we investigated the anticancer activity of TEOA against pancreatic cancer, and our results showed that TEOA significantly suppressed the proliferation and migration of pancreatic cancer cells. In addition, we found that TEOA preferentially targeted pancreatic cancer cells, while sparing low toxicity on non-malignant cells (Figure 9).

Mitochondria are important cellular organelles that can effectively utilize nutrients for energy production in the form of ATP (Giampazolias and Tait, 2016). Compared with normal cells, the survival of cancer cells relies more heavily on functional mitochondria (Deng and Haynes, 2017; Pustylnikov et al., 2018). Stimuli such as stress, hypoxia, and ischemia may lead to mitochondrial dysfunction, resulting in a decrease in MMP and ATP levels (Cadonic et al., 2016; Ham and Raju, 2017). Mitochondrial injury not only impairs cell homeostasis and biosynthesis but also plays a role in cell death, especially in cancer cells (Vara-Perez et al., 2019). Therefore, mitochondrial-targeted therapeutics have emerged as a promising method for tumor treatment (Weinberg and Chandel, 2015; Kalyanaraman et al., 2018). In our study, we collected evidence on mitochondrial damage, including morphological changes in mitochondria, decreased MMP, and reduced ATP production. These data suggest that TEOA induces mitochondrial dysfunction. Mitochondria are the principal source of ROS production, and damage to mitochondria may promote the overproduction of ROS (Panel et al., 2018). As expected, we subsequently detected an increase in ROS levels in TEOA-treated pancreatic cancer cells.

Autophagy is an important process in recycling cellular compounds and damaged organelles. A growing evidence demonstrates excessive ROS accumulation can induce autophagy through AMPK/mTOR pathway (Filomeni et al., 2015). In addition, oxidative stress acting as a major point in the activation of ATM protein, the core component of the DNA repair system, which is also involved in autophagy regulation (Xie et al., 2021). In this study, the results from western blotting, TEM and confocal microscopy indicated the occurrence of autophagy with the treatment of TEOA. To explore the specific mechanism of autophagy, we found that TEOA inhibited the activation of mTOR and its downstream target protein p70S6K/S6. Autophagy serves as a metabolic mechanism in response to cellular stress to promote cell survival (Galati et al., 2019). However, it can also induce type II programmed cell death (Liu et al., 2019). Previous studies have shown that targeting autophagy using natural compounds may provide therapeutic benefits for cancers (Deng et al., 2019; Yang et al., 2019). We further investigated the role of autophagy in TEOA-induced cytotoxicity in pancreatic cancer cells. The results revealed that inhibition of autophagy by 3MA and CQ or *ATG5* knockdown alleviated the cytotoxicity caused by TEOA, whereas inducing autophagy by rapamycin and EBSS aggravated cell death. These results indicate that ACD is involved in TEOA-induced pancreatic cell death.

ROS are well-known contributors to the inhibition of mTOR activation. In non-small cell lung cancer, increased ROS acts as the initial signal to inhibit the mTOR signaling pathway, thus resulting in the activation of ACD (Teng et al., 2019). In our study, to clarify whether TEOA-induced ACD is ROS-dependent, we investigated the rescue effects of the ROS scavenger NAC. As expected, NAC treatment successfully prevented ROS generation and restored cell viability. Moreover, western blotting results showed that NAC alleviated the inhibition of the mTOR signaling pathway, thus protecting pancreatic cancer cells from TEOA-induced ACD. Hence, our data implied that TEOA-induced ACD in pancreatic cancer cells was ROS dependent.

It is worth noting that drugs often play anti-cancer roles through more than one mechanism. Zhang et al. (2019) reported that β-Thujaplicin, a kind of natural bioactive product, exerted strong anti-HCC cancer ability via inducing both autophagic cell death and apoptosis. Apart from TEOA induced ACD, we found pan-caspase inhibitor Z-VAD-FMK also partially restored cell viability, indicating that apoptosis may also be involved in the TEOA-induced toxicity. This may be the reason why Z-VAD-FMK, CQ or 3MA couldn’t fully block the cell death induced by TEOA and merits further exploration. The crosstalk mechanism or relationship between apoptosis and autophagy cell death merits further exploration. Gemcitabine and cisplatin act as the mainstay of chemotherapeutic agents in the therapy of pancreatic cancer. However, acquired drug resistance and high toxicity lead to the discontinuation of therapy. Our recent study demonstrated that dihydroartemisinin could effectively optimize the antitumor activity of cisplatin, and significantly reduced its effective concentrations (Du et al., 2021). Therefore, it’s interesting to explore whether TEOA could be a promising adjuvant to improve the chemotherapy or overcome the chemoresistance of pancreatic cancer in the future. In conclusion, the above data demonstrated that TEOA inhibited the proliferation and migration of human pancreatic cancer SW1990 and PANC1 cells. In addition, TEOA provoked a cascade of alterations originating from the dysfunctional mitochondria, which induced ROS accumulation, followed by autophagy. Mechanistically, blocking the mTOR/p70S6K signaling pathway induced by TEOA leads to ACD in human pancreatic cancer cells. Our study, therefore, suggests that TEOA may be a promising therapeutic agent against human pancreatic cancer.

## Data Availability Statement

The original contributions presented in the study are included in the article/Supplementary Material, further inquiries can be directed to the corresponding authors.

## Author Contributions

JD, YW, and XT contributed to the conception and design of this study. CYa, YL, and WH acquired the data and drafted the manuscript. XW, JH, and CYu made the analysis. CZ and HW were responsible for the materials and methods part as well as English grammar of the whole manuscript. All authors contributed to manuscript revision, read, and approved the submitted version.

## Conflict of Interest

The authors declare that the research was conducted in the absence of any commercial or financial relationships that could be construed as a potential conflict of interest.

## Publisher’s Note

All claims expressed in this article are solely those of the authors and do not necessarily represent those of their affiliated organizations, or those of the publisher, the editors and the reviewers. Any product that may be evaluated in this article, or claim that may be made by its manufacturer, is not guaranteed or endorsed by the publisher.

## Supplementary Material

Supplementary Figure 1 TEOA induced selective cytotoxicity of human pancreatic cancer cells. **(A)** Human normal pancreatic cells HPDE6-C7 cells, pancreatic cancer cells SW1990 and PANC1 cells were treated with various concentrations of TEOA (0–60 μM) for 24 h. The cell viability was detected using CCK8 assays. **(B)** HPDE6-C7 cells and SW1990 cells were treated with various concentrations of TEOA (0–50 μM) for 12 h. The cell viability and cytotoxicity were detected using Calcein/PI cell viability/cytotoxicity assay (^★★^*p* < 0.01).

Supplementary Figure 2 Transwell assay of HPDE6-C7 cells treated with 0–25 μM TEOA for 24 h.

Supplementary Figure 3 To assess intracellular ROS production, TEOA treated SW1990 and HPDE6-C7 cells were loaded with DCFH-DA, mitoSOX probe for 30 min followed by confocal laser microscope (Scale bars: 25 μm).

Supplementary Figure 4 **(A)** SW1990 cells were treated with TEOA alone or in combination with 0.5 mM NAC, and then mitochondria membrane potential levels were measured by flow cytometry after 8 h. **(B)** Statistical analysis of relative mitochondria membrane potential level and the results were presented as mean ± SD from three independent experiments (^★★^*p* < 0.01).

Supplementary Figure 5 Human normal pancreatic cells HPDE6-C7 cells, pancreatic cancer cells SW1990 and PANC1 cells were treated with TEOA at the indicated concentration of TEOA for 8 h and the ratio of LC3I and LC3II were detected by western blot.

Supplementary Figure 6 SW1990 cells were treated with/without TEOA (40 μM) for 8 h, and autophagic vacuoles were observed using TEM. Scale bar: 2 μm (left) or Scale bar: 0.5 μm (right).

Supplementary Figure 7 After exposure to TEOA for 8 h, pancreatic cancer cells were collected to measure the level of p-AMPK and AMPK, GAPDH used as a loading control.

Supplementary Figure 8 Statistical analysis of Figure 8E and the results were presented as the mean ± SD from three independent experiments (^★★^*p* < 0.01, ^★★★^*p* < 0.001).

Supplementary Figure 9 Indicated treated PANC1 cells were harvested and mTOR signaling pathway was determined by western blot.

Supplementary Figure 10 SW1990 cells were treated with TEOA alone or combined with CQ (25 μM), Z-VAD-FMK (5 μM), respectively, and CCK8 test was used to assess cell viability (^★^*p* < 0.05).

Supplementary Figure 11 SW1990 cells were treated with TEOA for 12 h, and the cell apoptosis was detected using Annexin V-FITC/PI apoptosis kit by flow cytometry.
