# Supplementary figures and images for: TEOA Promotes Autophagic Cell Death via ROS-Mediated Inhibition of mTOR/p70S6k Signaling Pathway in Pancreatic Cancer Cells
Source: Front Cell Dev Biol. 2021 Oct 6;9:734818. doi: 10.3389/fcell.2021.734818 (PMC8526869; doi:10.3389/fcell.2021.734818)

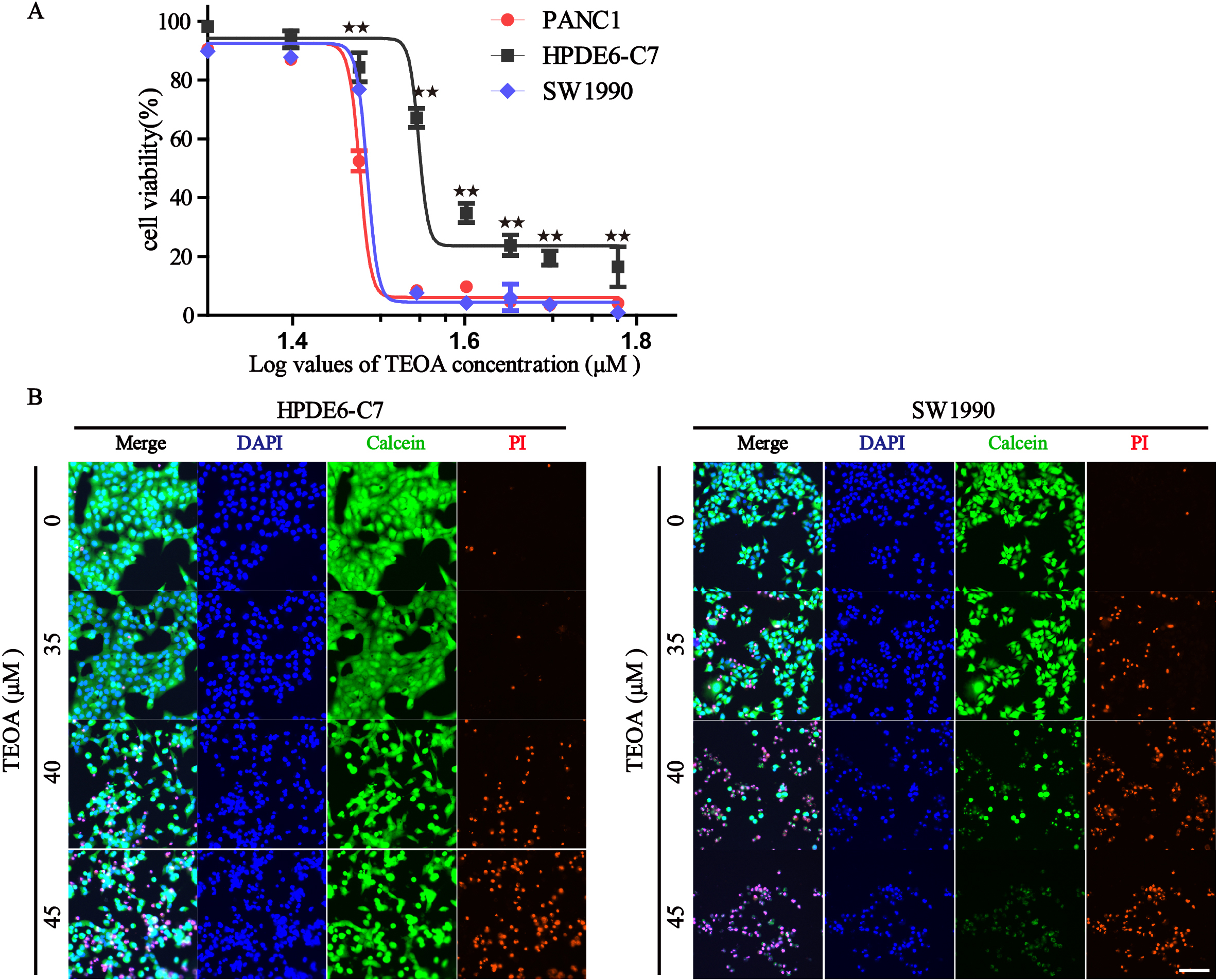

Supplement: Supplementary Figure 1 — TEOA induced selective cytotoxicity of human pancreatic cancer cells. (A) Human normal pancreatic cells HPDE6-C7 cells, pancreatic cancer cells SW1990 and PANC1 cells were treated with various concentrations of TEOA (0–60 μM) for 24 h. The cell viability was detected using CCK8 assays. (B) HPDE6-C7 cells and SW1990 cells were treated with various concentrations of TEOA (0–50 μM) for 12 h. The cell viability and cytotoxicity were detected using Calcein/PI cell viability/cytotoxicity assay (★★p < 0.01). [file Image_1.JPEG]

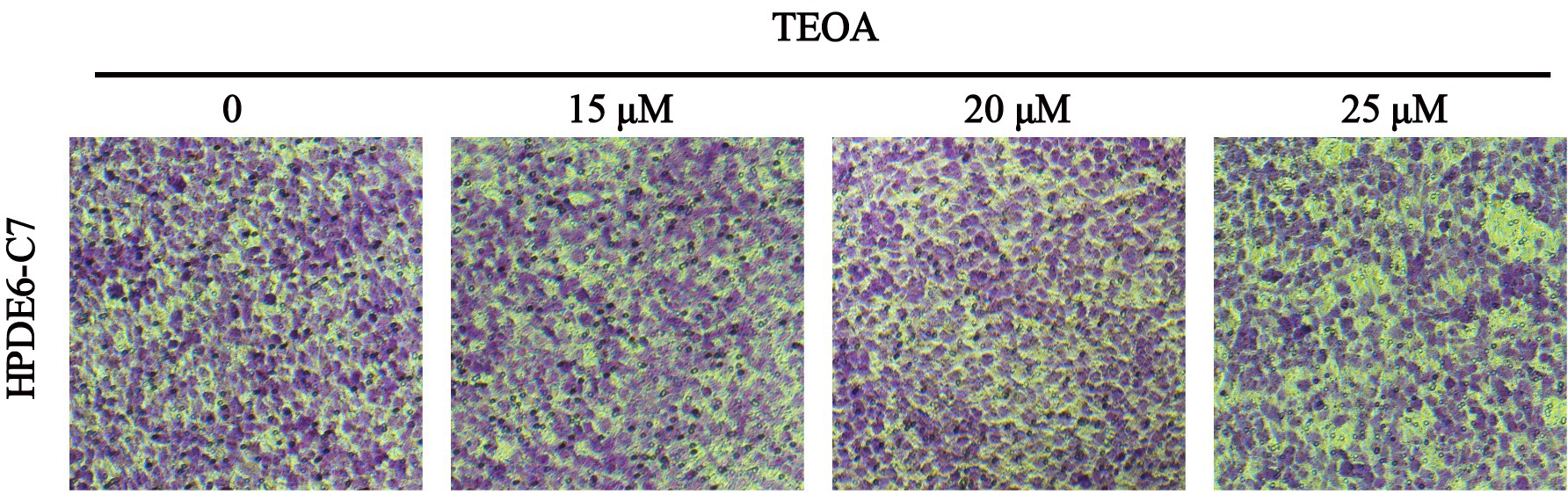

Supplement: Supplementary Figure 2 — Transwell assay of HPDE6-C7 cells treated with 0–25 μM TEOA for 24 h. [file Image_2.JPEG]

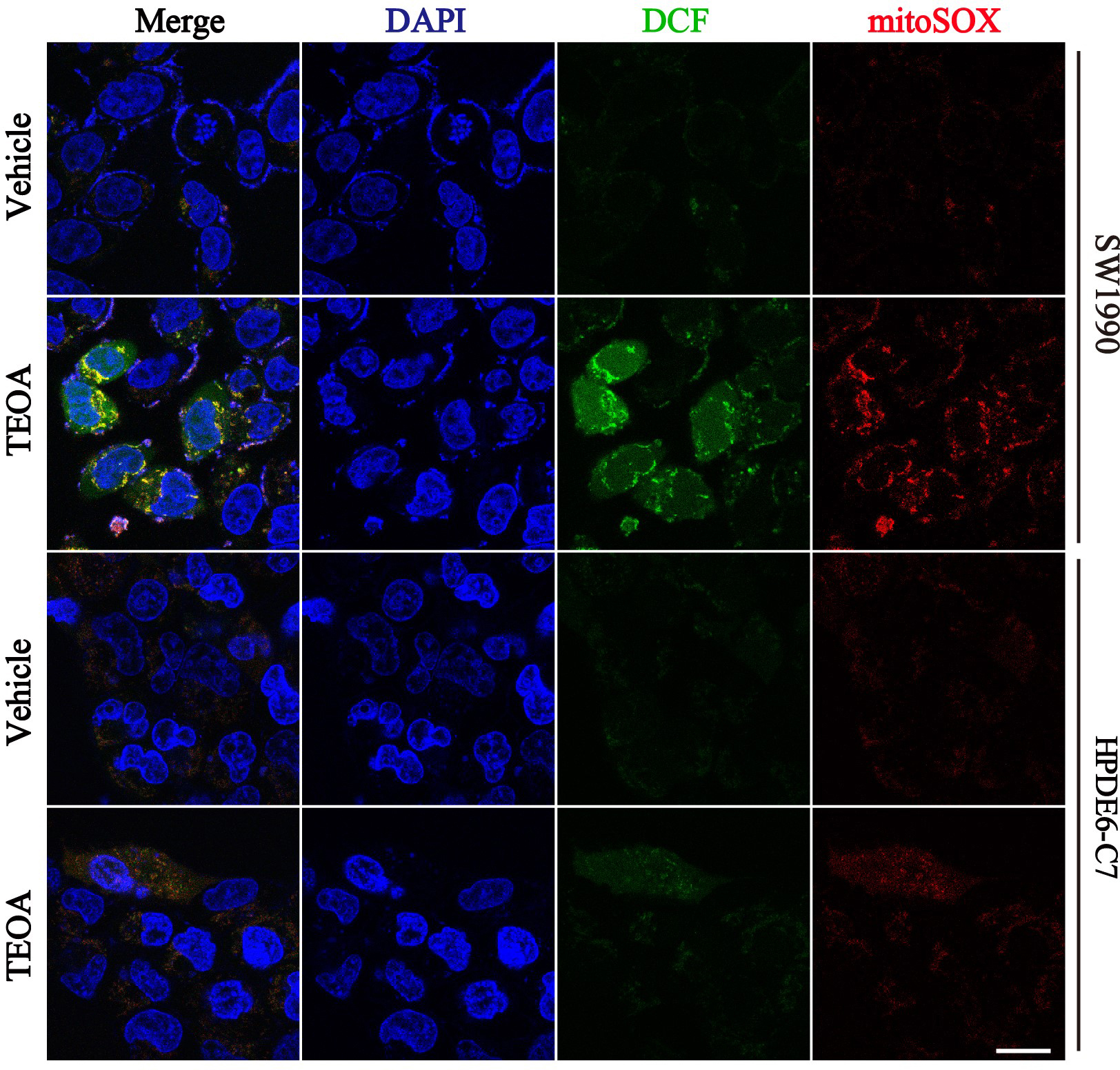

Supplement: Supplementary Figure 3 — To assess intracellular ROS production, TEOA treated SW1990 and HPDE6-C7 cells were loaded with DCFH-DA, mitoSOX probe for 30 min followed by confocal laser microscope (Scale bars: 25 μm). [file Image_3.JPEG]

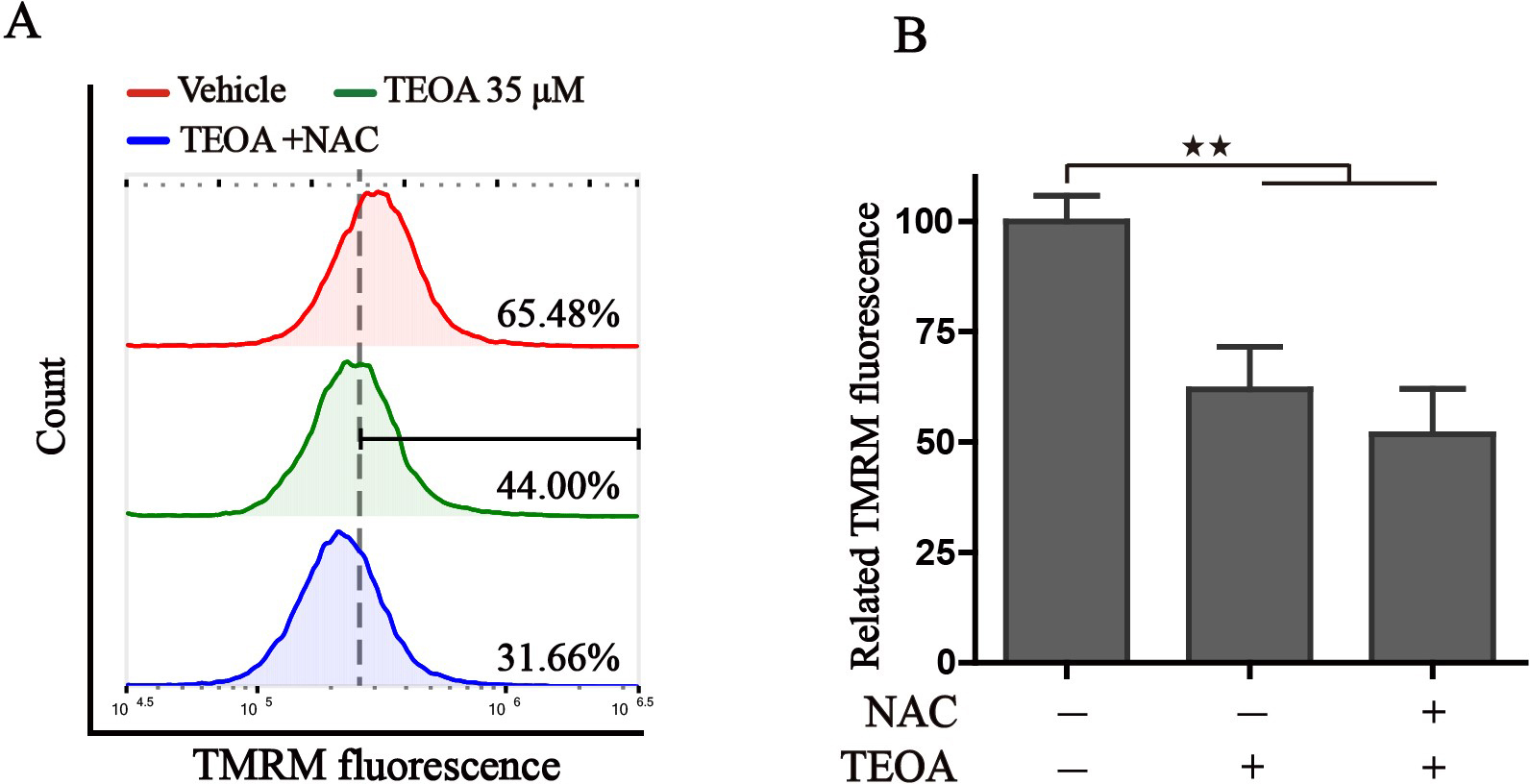

Supplement: Supplementary Figure 4 — (A) SW1990 cells were treated with TEOA alone or in combination with 0.5 mM NAC, and then mitochondria membrane potential levels were measured by flow cytometry after 8 h. (B) Statistical analysis of relative mitochondria membrane potential level and the results were presented as mean ± SD from three independent experiments (★★p < 0.01). [file Image_4.JPEG]

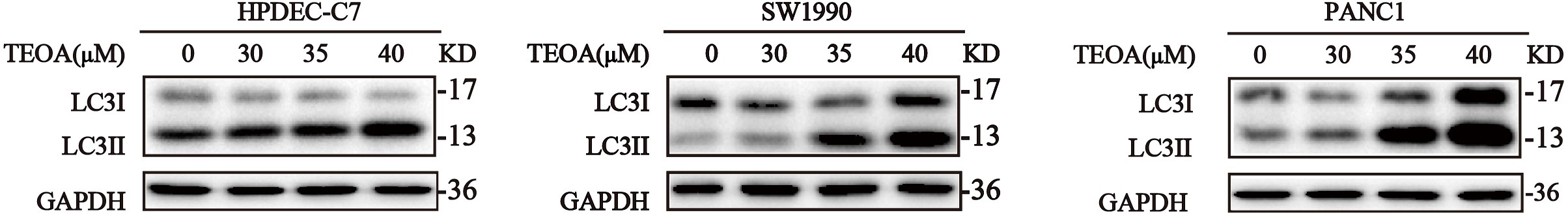

Supplement: Supplementary Figure 5 — Human normal pancreatic cells HPDE6-C7 cells, pancreatic cancer cells SW1990 and PANC1 cells were treated with TEOA at the indicated concentration of TEOA for 8 h and the ratio of LC3I and LC3II were detected by western blot. [file Image_5.JPEG]

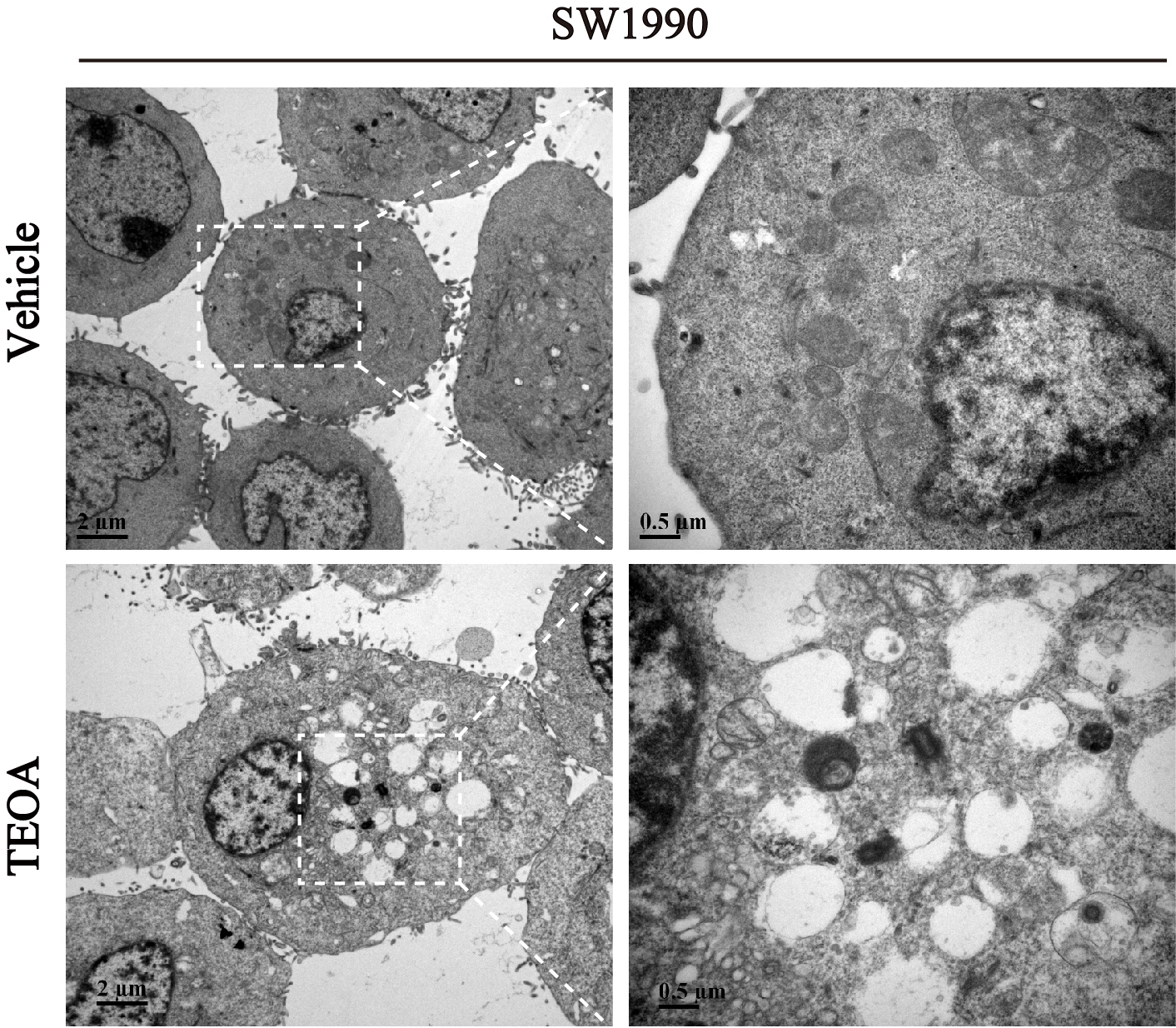

Supplement: Supplementary Figure 6 — SW1990 cells were treated with/without TEOA (40 μM) for 8 h, and autophagic vacuoles were observed using TEM. Scale bar: 2 μm (left) or Scale bar: 0.5 μm (right). [file Image_6.JPEG]

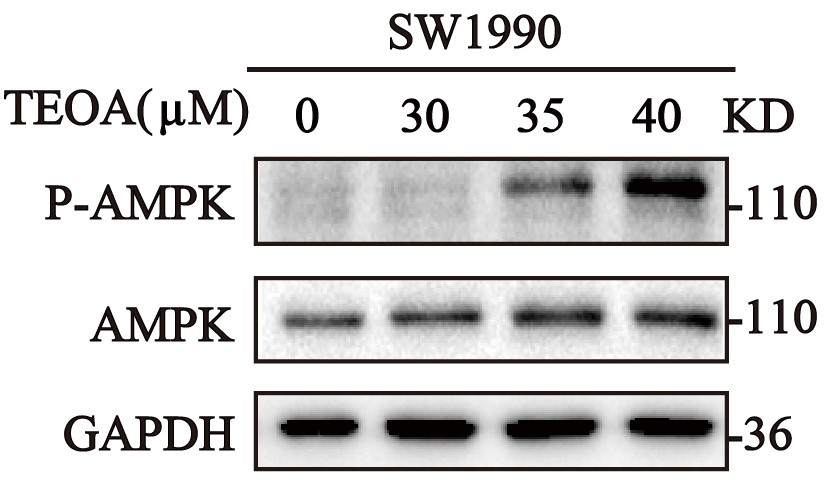

Supplement: Supplementary Figure 7 — After exposure to TEOA for 8 h, pancreatic cancer cells were collected to measure the level of p-AMPK and AMPK, GAPDH used as a loading control. [file Image_7.JPEG]

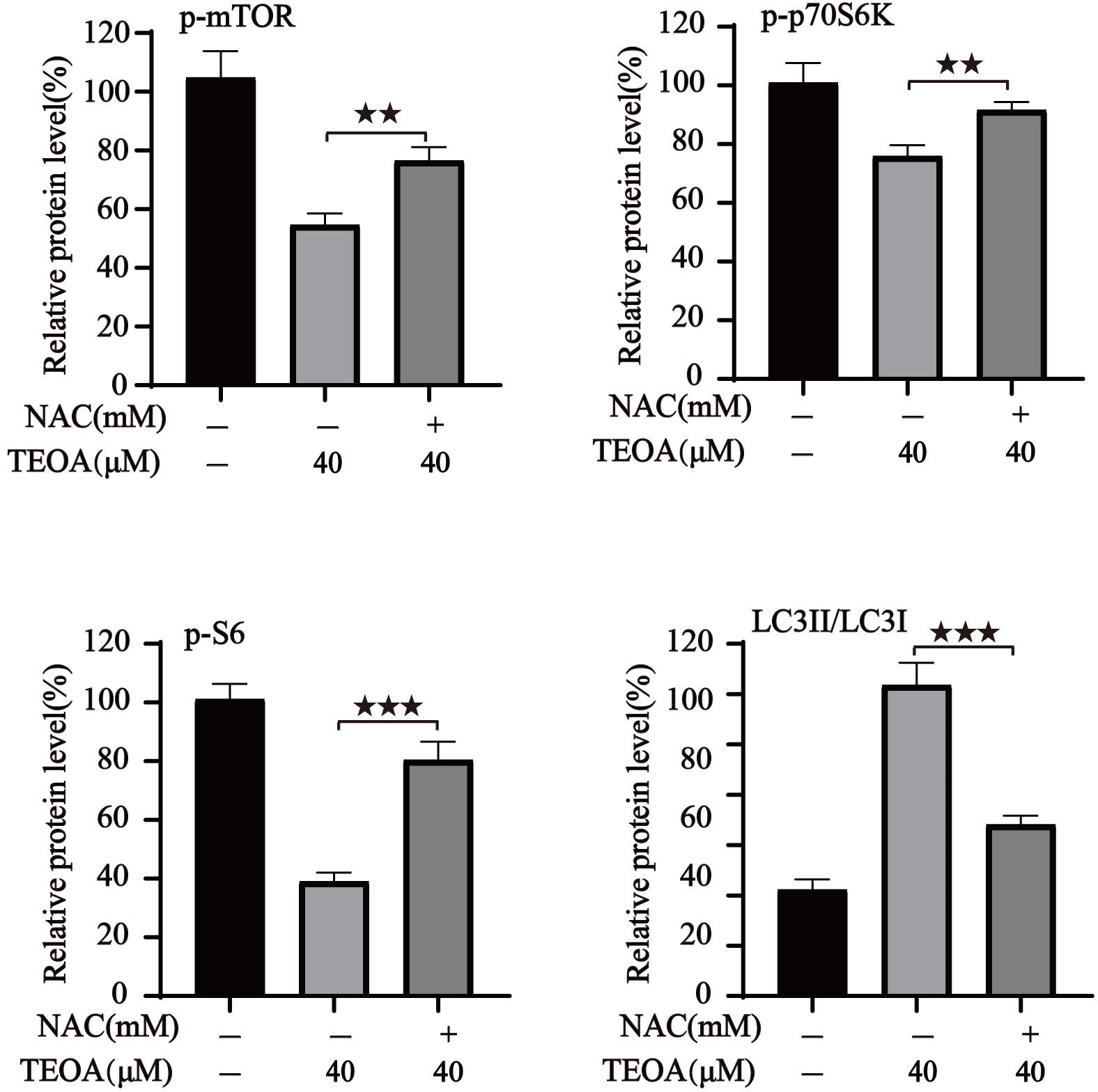

Supplement: Supplementary Figure 8 — Statistical analysis of Figure 8E and the results were presented as the mean ± SD from three independent experiments (★★p < 0.01, ★★★p < 0.001). [file Image_8.JPEG]

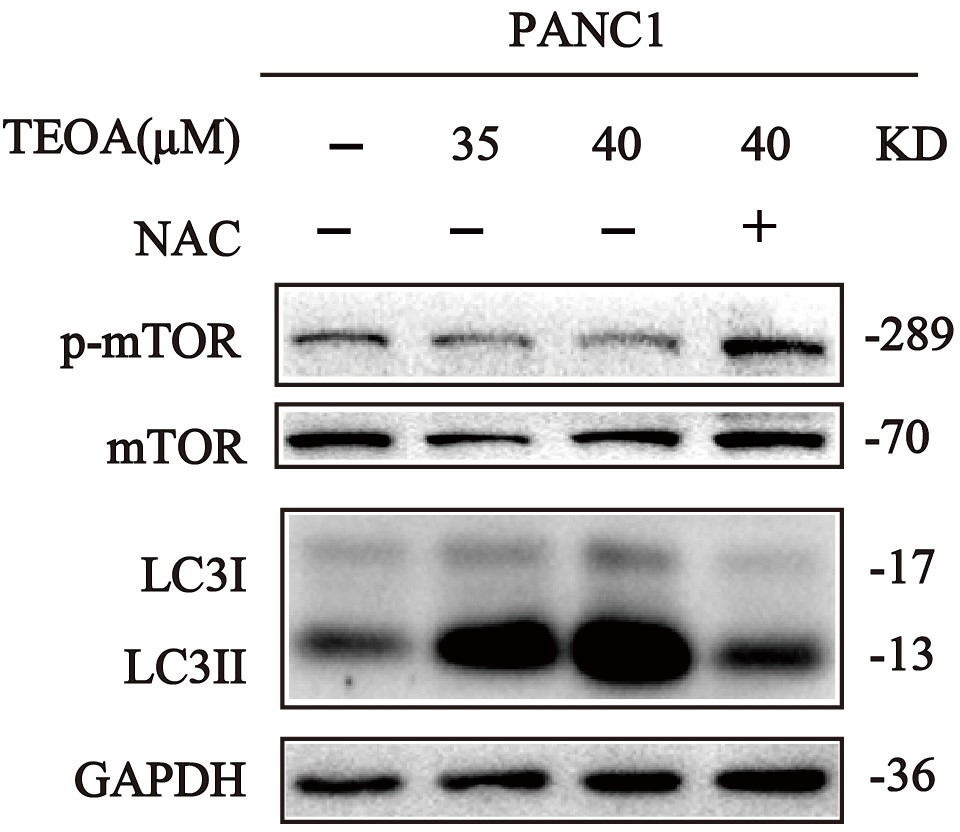

Supplement: Supplementary Figure 9 — Indicated treated PANC1 cells were harvested and mTOR signaling pathway was determined by western blot. [file Image_9.JPEG]

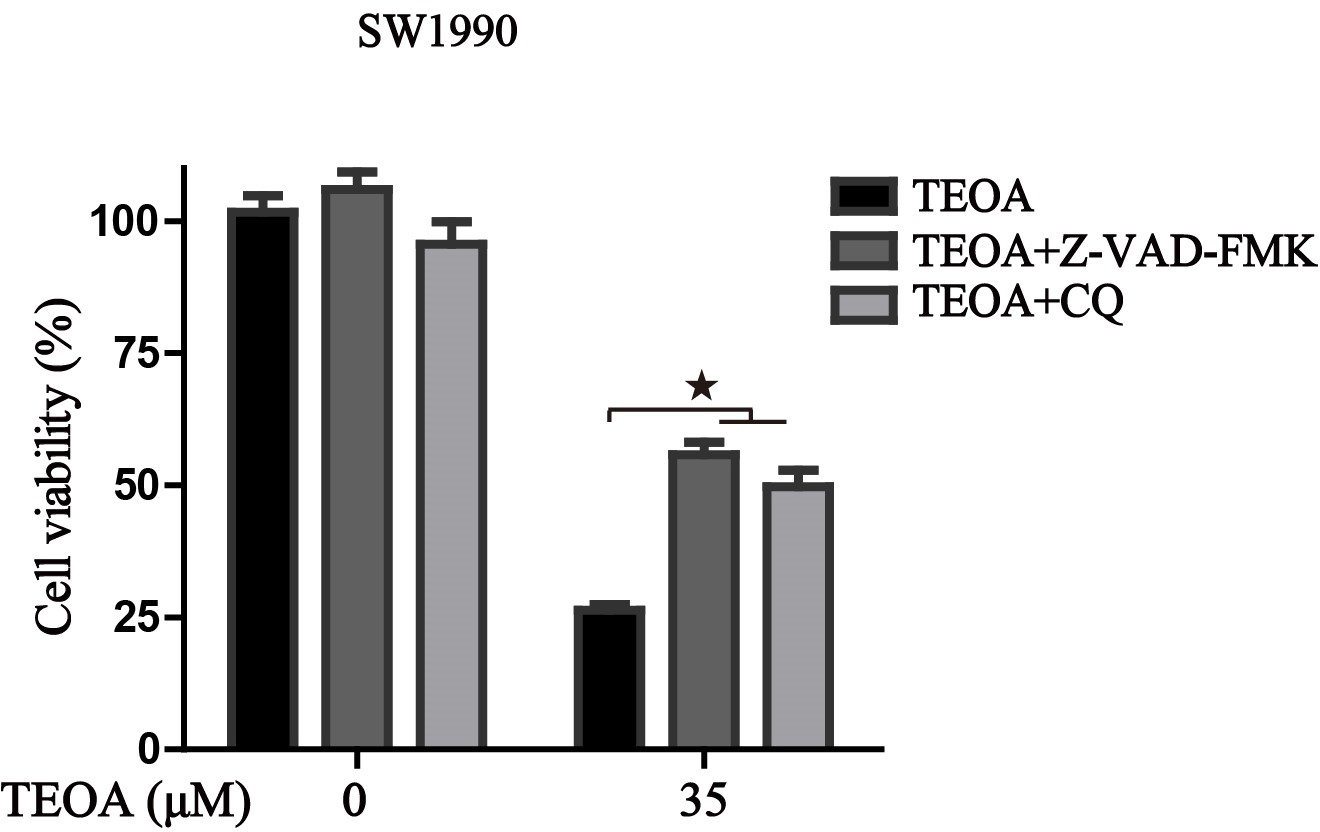

Supplement: Supplementary Figure 10 — SW1990 cells were treated with TEOA alone or combined with CQ (25 μM), Z-VAD-FMK (5 μM), respectively, and CCK8 test was used to assess cell viability (★p < 0.05). [file Image_10.JPEG]

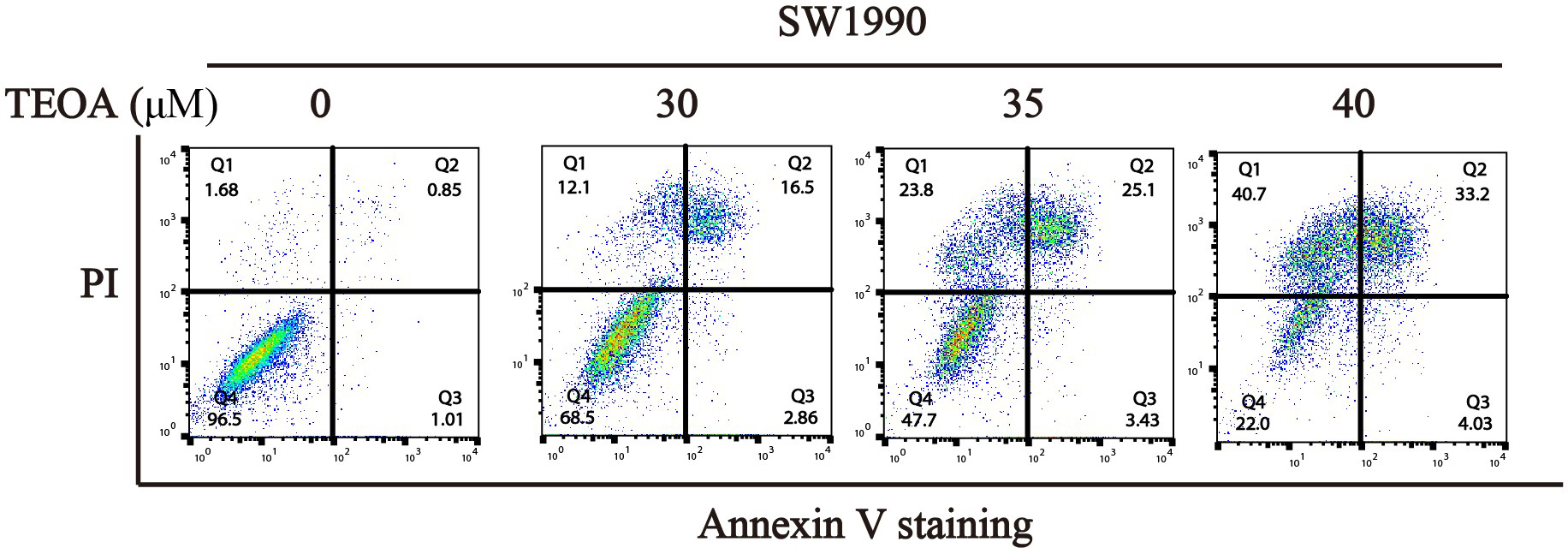

Supplement: Supplementary Figure 11 — SW1990 cells were treated with TEOA for 12 h, and the cell apoptosis was detected using Annexin V-FITC/PI apoptosis kit by flow cytometry. [file Image_11.JPEG]
